# Hemorrhagic Malarial Fever

**Published:** 1877-07-20

**Authors:** L. A. Guild

**Affiliations:** Georgia


### HEMORRHAGIC MALARIAL FEVER.

By L. A. GUILD, M.D., of Ga.

This disease, in the South Atlantic and Gulf States, is often called swamp fever, country yellow fever, icteric yellow fever. It makes its appearance, usually, in the malarial districts, in the latter, part of summer, and during the fall months.

It begins with a chill, and is followed by vomitings of a green color, and not unlike arseniate of copper. Often, in the latter course of this fever, black vomit sets in, and stools of a similar nature, sometimes become bloody, the urine yellow, but oftener it has the appearance of blood and is highl ycharged with *cholephrrhin*, but contains no fibrin.

The sick have, almost invariably from the first day, a continued fever, irregularity of pulse, drowsiness, with delirium, and *jaundice*. An icteral suffusion is soon spread over the whole body, and the skin becomes of a deep orange hue. Anatomical lesions—softening of the gastric mucous membrane, alteration of the color of the liver, which, with the spleen and kidneys, is hypertrophied, cholecchysis in the blood and secretions.

We have good reason to believe that the toxæmic impression of malaria, and its effects upon the nerve-centres, (either of organic or of animal life,) are primary, and congestion of the liver, with redundancy of bile, the secondary cause.

This hemorrhagic malarial fever is becoming more prevalent throughout the South with every autumnal season, and has thus far been more rebellious to treatment, and proved a great deal more fatal to its victims, than the pure epidemic yellow fever of the coast.

I have had to encounter a great many cases of this fever during the last eighteen years, while practicing medicine in Southwestern Georgia. Saw a Baldwin, M.D., graduate of the New Orleans Medical College, aged 30, health previous to attack good, found him very sick, hemorrhagic malarial fever of two days standing, vomiting dark green matter, frequent and aqueous bloody stools, micturition often, urine copious and looked like blood, skin yellow as an orange, pulse 135, weak. His case was truly alarming. He had taken, previous to my seeing him, by his own prescription, freely of sulphate quinine, calomel, mucilage, gum arabic, spirits turpentine, anodynes, etc. Prescribed—

R. Sulph morphia............ii gr.
Creasote......................x gtt.
Acetic acid.....................xx gtt.
Aquæ..........................i fl. oz.

M. S. one teaspoonful every hour until the vomiting becomes less.

Also—

R. Tinc. ferri. chlor. and
sulphuric acid, dilut...aa. fl. dr. ij
Aquæ.....................fl. oz. iv

M. S. a tablespoonful every two hours.

In six hours after commencing this treatment, the doctor had retained sufficient of the medicine to render him much more comfortable. A similar treatment was continued, but not given so often, for forty-eight hours, (most of which time I was with him), when he was much better, the vomiting and sanguineous discharges had ceased, the urine was changed to a deep yellow color and less in quantity; pulse 90. In eight or ten days he fully recovered.

In my experience with this fever, there never was a more fatal error in administering medicine to the sick than to give calomel, cathartics, emetics or quinine in this disease, before the dangerous phenomena are removed. Notwithstanding malaria is a cause, and the pyrexiæ is not sthenic, *quinine is contra-indicated*, as it increases the gastric irritation, and in large doses acts as a cholagogue, which in this condition invariably aggravates the morbid symptoms. Where there was a latent pre-disposition I have known this fever to be brought on by the quinine, which was given in large doses for intermittent fever, and the same has often occurred after taking calomel and other active cathartics. A boy, aged 6, had light tertian intermittent; his mother gave him a dose of may apple pills; in thirty hours after, this fever set upon him in a fearful form.

I prefer tinc. opii. to morphia in this fever, and have been quite successful with the following prescriptions:

R. Tinc. opii................ii fl. oz.
Carbolic acid..............iv gtt.

M.—S. Thirty drops every two hours.

Sometimes I give—

R. Sulph. morphia............ii gr.
Creosote.....................x gtt.
Acetic acid...................xx gtt.
Aquæ..........................i fl. oz.

M.—S. A teaspoonfull every hour until the system is brought under the effects of the medicine (being cautious not to produce coma.) Should the stomach reject everything taken into it, I often use—

R. Tinc. opii...................i dr.

Carbolic acid.............. ii drops.

Tinc. camphor...............xx drops
Water.......................ii oz.
M.—Give per enema.

Or, often use $\frac{1}{8}$ to $\frac{1}{6}$ grain morphia hypodermically, until the stomach will tolerate the medicine.

We know that in most complaints of the liver and its appendages, that opium is seldom beneficial; but in this condition it is indicated. It not only acts as a palliative, but it stops the vomiting, checks the excretion and secretion of bile, which soon removes all the morbid symptoms. The patient is to be kept under some impression of the opiate during the violence of the disease.

I also find great benefit, when the tongue is deep red, from—

R. Tinct. ferri chlor. and dilut. sulpuric acid..........aa. f. dr. ii;
Aquæ puræ..............f. 4 oz. M.

S. A tablespoonful every two hours; after a few doses have been taken, every four or five hours. When the tongue is pallid, with dirty white, pasty coating, I prescribe instead of the acid treatment—

R. Carb. ammonia.............dr. i.
Chlor. Potash.............gr. xl.
Water....................f. oz. iv. M.

S. A tablespoonful every two hours; this may not be oftener than once in four or five hours after it has been given five or six times every two hours.

The patient must be put upon a very light diet, and the stomach and bowels must be kept as quiet as possible. As a restorative, after the urgent symptoms have passed off, I have found the fluid ext. of chionanthus virginica, given in teaspoonful doses three times a day, acts well in these cases, and in jaundice generally. Fowler's solution, also, in four or five drop doses, once every four or five hours, is efficient for the same purpose.

[The above is the cure for hemorrhagic malarial fever promised by Dr. G. in our April number. We trust the profession will test the method of treatment suggested in this hitherto formidable and unmanageble disease. —Ed.]

